# Lifetime Prevalence of Recurrent and Persistent Depression: A Scoping Review of Epidemiological Studies

**DOI:** 10.2174/0117450179372815250516102324

**Published:** 2025-05-23

**Authors:** Evgeny Kasyanov, Yana Yakovleva, Maria Khobeysh, Ekaterina Gerasimchuk, Galina Mazo

**Affiliations:** 1 Department of Social Neuropsychiatry, V. M. Bekhterev National Medical Research Center for Psychiatry and Neurology, Saint-Petersburg, Russia; 2 Department of Integrative Pharmaco-Psychotherapy, V. M. Bekhterev National Medical Research Center for Psychiatry and Neurology, Saint-Petersburg, Russia

**Keywords:** Recurrent depression, Persistent depression, Dysthymia, Intermittent major depression, Lifetime prevalence, PRISMA

## Abstract

**Background:**

Differing conceptualizations of recurrent and persistent depression in the Diagnostic and Statistical Manual of Mental Disorders (DSM) and International Classification of Diseases (ICD) lead to diagnostic inconsistencies. This scoping review analyzed epidemiological studies on the lifetime prevalence of recurrent and persistent depression in the general population.

**Methods:**

Following PRISMA-ScR guidelines, we searched MEDLINE and the Russian Science Citation Index without time or language restrictions. Inclusion criteria were original epidemiological studies of the general population reporting lifetime prevalence of recurrent or persistent depression, using DSM-III/IV/5 or ICD-9/10/11 criteria.

**Results:**

Only three studies on recurrent depression were identified – from Switzerland, the USA, and Hungary – showing a consistent lifetime prevalence of 10.3% to 10.5%. In contrast, major depressive disorder had a lifetime prevalence of 1.5 to 2.5 times higher in these studies. Dysthymia and persistent depression showed lifetime prevalences ranging from 1.1% to 6.4% and 1.6% to 18%, respectively. Women represented about two-thirds of cases of both recurrent and persistent depression.

**Conclusion:**

The underrepresentation of recurrent depression may stem from the DSM's dominant influence in psychiatric diagnostics. Our findings highlight the need for refined diagnostic criteria and more comprehensive epidemiological studies that separately identify recurrent and persistent depression.

## 1 INTRODUCTION

The Diagnostic and Statistical Manual of Mental Disorders (DSM) and the International Classification of Diseases (ICD) conceptualize recurrent and persistent depression differently. In ICD-10 and ICD-11, a distinction is made between a single depressive episode and recurrent depressive disorder, the latter implying the presence of two or more depressive episodes during one's life with mandatory periods of remission between them (*i.e.*, several months without significant mood disturbances) [1]. The DSM-5 does not place such a fundamental focus on the recurrent course of depression; major depressive disorder can manifest as either a single depressive episode or multiple episodes over a lifetime [2]. Recurrence and severity of depressive episodes are indicated separately in the DSM-5, which is why these aspects are often not considered in most epidemiological, clinical, and basic studies that use the DSM framework. However, relapses after the first depressive episode occur in up to 75% of cases, according to various estimates [3]. Additionally, recurrent depression is characterized by a greater genetic predisposition and a higher frequency of family history of mood disorders and is also associated with concomitant anxiety disorders and adverse childhood experiences [4, 5].

Notably, about 30% of all depressions have a persistent course [6, 7], which is why they are increasingly distinguished from recurrent forms [8, 9]. To characterize states with a depressed mood lasting for two years or more, the DSM-5 identifies persistent depressive disorder as a separate diagnosis. This includes specifiers for pure dysthymia (symptoms not reaching the level of a major depressive episode), persistent major depressive disorder (symptoms reaching the level of a major depressive episode), and intermittent major depressive disorder (“double depression”: dysthymia combined with recurrent depression) [2]. An important feature is that persistent depressive disorder concerns only unipolar mood disorders; therefore, persistent depressive episodes within the framework of bipolar disorder are not conceptualized, and cyclothymic disorder is classified as a separate diagnosis. In ICD-11, dysthymic and cyclothymic disorders are presented as separate diagnoses and a persistent course is indicated as a specifier for any mood episodes, allowing its use for mood disorders of any polarity [1].

All these subtle differences in the diagnostic manuals of the DSM and the ICD give rise to many inconsistencies in the description of recurrent and persistent depression. Nevertheless, in scientific research worldwide, the DSM is considered the “gold standard” for diagnosis, not necessarily due to the classification itself but because of the widely used structured diagnostic interviews developed based on it [10]. This results in the conventional nature of the DSM being combined with the subjectivity of the developers of diagnostic interviews, who do not always take into account all the specifiers and clinical features of recurrent and persistent depression. Consequently, two significant biases can be observed in scientific research: first, an insufficient focus on recurrent depression; second, an insufficient focus on persistent depression within the framework of bipolar disorder, especially in cases with a continuous course [11-14].

Therefore, numerous gaps and controversial diagnostic issues exist in the study of recurrent and persistent depression. Currently, we have not found systematic studies on the prevalence of these disorders nor critical articles analyzing the methodologies of such epidemiological studies. For this reason, the aim of this scoping review was to conduct an exploratory analysis of epidemiological studies on the lifetime prevalence of recurrent and various forms of persistent depression in the general population.

## 2 MATERIALS AND METHODS

This review was conducted following the extended PRISMA (Preferred Reporting Items for Systematic Reviews and Meta-Analyses) guidelines for scoping reviews [15]. The protocol for this study was not registered in any public sources. The protocol can be obtained by sending a justified request to the corresponding author.

### 2.1 Search Strategy

We searched the electronic databases MEDLINE and the Russian Science Citation Index without time or language restrictions, using the following keywords: ((recurrent depressive disorders[Title /Abstract]) OR (recurrent major depressive disorder[Title/ Abstract])) AND ((dysthymia[Title/ Abstract]) OR (persistent depressive disorder[Title/ Abstract]) OR (dysthymic disorder[Title/ Abstract]) OR (chronic major depressive disorder[Title/ Abstract]) OR (dysthymic syndrome[Title/ Abstract])) AND ((epidemiology[Title/ Abstract]) OR (prevalence[Title/ Abstract])). Additionally, we conducted a manual search using the reference lists of recent systematic reviews and meta-analyses [16-20].

### 2.2 Eligibility Criteria

The inclusion criteria were as follows: (1) original epidemiological studies of the general population; (2) studies on the prevalence of recurrent depression and various forms of persistent depression; (3) studies using diagnostic criteria for mood disorders from DSM-III, DSM-IV, DSM-5, or ICD-9, ICD-10, or ICD-11. Studies were excluded if: (1) there was significant overlap in the populations studied (in such cases, we selected the study with the largest sample size); (2) the study was not conducted on the general population; (3) the authors did not report that a diagnostic interview was conducted with participants to confirm or rule out a diagnosis of a mental disorder.

### 2.3 Review Strategy

Data were collected independently by three authors (YVY, MAK, and ESG) and confirmed by the other two authors of the review (EDK and GEM). A descriptive analysis of the selected studies was performed. The following data were extracted from the included articles: title, authors, year of publication, country, diagnostic method, sample sizes, and prevalence of the studied disorder in the general population (including among women and men). Additionally, other important observations related to the methods and results of each individual article were recorded. Data extracted from the publications were compiled into a table. The studies included in the review were not critically evaluated, and statistical methods for data analysis were not used.

### 2.4 Data Synthesis

Our analytical approach involved a descriptive analysis of the included studies. Given the wide variety of terms in the scientific literature, we decided to use the following integral concepts to describe the results of this review: (1) **recurrent depression** corresponds to recurrent depressive disorder in ICD-11 and major depressive disorder with a specifier for recurrence in DSM-5; (2) **dysthymia** corresponds to dysthymic disorder in ICD-11 and persistent depressive disorder with a specifier for pure dysthymia in DSM-5; (3) **persistent depression** corresponds to the specifier for a persistent course for depressive episodes of any polarity in ICD-11; (4) **intermittent depression** corresponds to persistent depressive disorder with a specifier for intermittent major depressive disorder in DSM-5 (“double depression”). Thus, in this study, we imply that persistent depression can also be observed in bipolar disorder.

From the original studies, we extracted lifetime prevalence rates of recurrent and persistent depression in the general population, including sex-specific estimates for women and men. Additionally, we noted the year and country of publication, as well as the diagnostic system used in each study.

## 3 RESULTS

### 3.1 Selection of Sources of Evidence

**Fig. (1):**
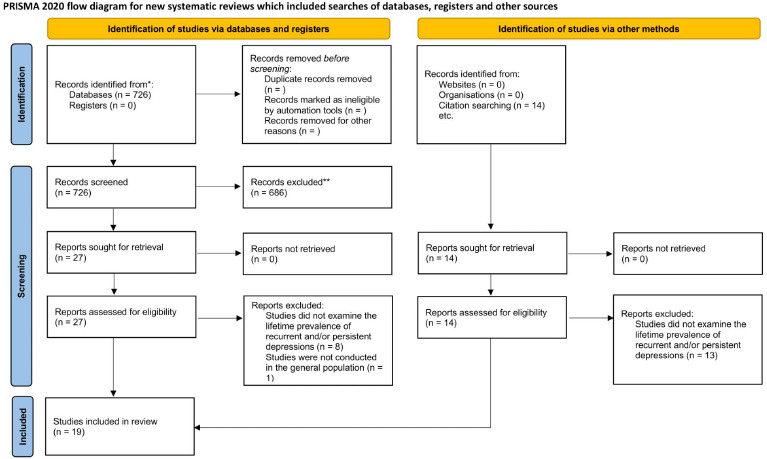
PRISMA 2020 flow diagram for systematic reviews resulting from a search of the database and other sources for a scoping review.

The search query identified 740 publications. After reviewing the titles and abstracts, 686 publications were excluded as not relevant to the topic of the review (reasons for exclusion at this stage were not specified for each source). Upon examining the full texts of 41 articles, we included 19 publications in the scoping review [6, 21-38]. The primary reason for exclusion from the analysis was that the studies were not conducted in the general population or did not examine the lifetime prevalence of recurrent and/or persistent depression (Fig. **1**).

### 3.2 Characteristics of Sources of Evidence

The articles selected for the review were published between 1989 and 2017. All publications included original epidemiological studies. Geographically, 8 studies were conducted in the USA [22, 24, 25, 27-31], 3 in Germany [33, 35, 37], and 1 each in Switzerland, Hungary, the Netherlands, Austria, Italy, France, Iran, Canada, and New Zealand [6, 21, 22, 26, 32, 34, 36, 38]. Recurrent depression was studied in only 3 epidemiological studies (total sample size n=18,848) [21-23], while dysthymia was studied in 17 (n=132,062) [6, 20, 22-33, 35-37], persistent depression in 4 (n=60,062) [6, 21, 28, 35], and intermittent depression in 1 (n=7,667) [31]. All epidemiological studies included in this review were conducted based on various versions of the DSM. The characteristics of the studies included in the review, as well as their main results, are presented in Tables **1** and **2**.

### 3.3 Results of Individual Sources of Evidence

The results of the three epidemiological studies that examined recurrent depression were very similar. As shown in Table **1**, its lifetime prevalence ranged from 10.3% to 10.5% in the general population. The proportion of women among all patients with recurrent depression ranged from 69.1% to 75.9%. In contrast, the prevalence of *major depressive disorder,* according to the DSM, among the included studies was more variable, ranging from 14.4% to 27.9% in the general population.

As shown in Table **2**, the lifetime prevalence of dysthymia ranged from 1.1% to 6.4%, with the proportion of women among all patients with dysthymia ranging from 58.1% to 75.8%. In turn, the lifetime prevalence of persistent depression ranged from 1.6% to 18%, with the proportion of women among all patients with persistent depression ranging from 65.8% to 70.2%. Only one study reported the prevalence of intermittent depression in the general population, which was 3.4%, but without data on the prevalence among women and men.

## 4 DISCUSSION

In our exploratory analysis of epidemiological studies on the lifetime prevalence of recurrent and various forms of persistent depression, we identified several important observations. First, there are only three epidemiological studies on recurrent depression from the general populations of Switzerland, the USA, and Hungary, which are far fewer than the number of studies on dysthymia and persistent depression. The exception is intermittent depression, for which only one epidemiological study has been conducted. However, the nosological validity of “double depression” remains a subject of debate [9, 39]. Second, the lifetime prevalence of recurrent depression in the general population remained stable across all three studies, ranging from 10.3% to 10.5%. In contrast, the prevalence of major depressive disorder in these same studies was 1.5 to 2.5 times higher. Third, the lifetime prevalence of dysthymia and persistent depression ranged from 1.1% to 6.4% and from 1.6% to 18%, respectively. It is worth noting that such a high prevalence of persistent depression was found only in one study [21], while in the others, it did not exceed 4.5%. Fourth, the proportion of women with recurrent and persistent depression, as well as dysthymia, was quite stable, accounting for about two-thirds of all cases. Finally, all epidemiological studies included in this review were based on various versions of the DSM and were predominantly conducted in populations from North America and Western Europe, with the exception of two studies from New Zealand and Iran.

The lack of attention to recurrent depression neglects not only chronic episodic depressions, which contribute significantly to the disease burden, but also underestimates non-chronic depressions with a favorable prognosis, often requiring no antidepressant therapy [3]. This distortion stems primarily from the dominant position of the DSM in psychiatric science and its use of the nonspecific construct of *major depressive disorder*.

The diagnostic validity of *major depressive disorder* has been widely criticized in the scientific literature. Introduced in DSM-III (1980), *major depressive disorder* remains largely unmodified and encompasses a heterogeneous mix of depressive conditions [40]. Historically, the concept of *manic-depressive illness* included all recurrent mood disorders, regardless of polarity (depressive, manic, or mixed episodes) [41-43]. However, DSM-III reclassified these conditions, merging recurrent depression, reactive depression, and persistent severe depression (*e.g.*, involutional melancholia) into the single construct of major depressive disorder [40-46]. This shift eliminated the diagnostic significance of recurrence and persistent course, treating a single depressive episode – whether isolated, persistent, or the onset of bipolar disorder – as part of major depressive disorder.

While DSM-5 granted independent nosological status to persistent depressions, recurrent depressions remain without diagnostic independence. Notably, DSM-5 restricts persistent depression to unipolar depression, unlike DSM-III-TR, which allows this specifier for bipolar disorder [2]. In contrast, ICD-11 permits persistent depression in both unipolar and bipolar disorders, a distinction that should inform future research [1]. Although a direct comparison of the prevalence of recurrent and persistent depression under ICD and DSM criteria has not been conducted, it is reasonable to assume that prevalence estimates for recurrent depression would be similar across both systems. However, this may not hold true for persistent depression, given the differing diagnostic approaches in DSM-5 and ICD-11. These discrepancies highlight the need for further research to clarify how diagnostic criteria influence prevalence estimates and to ensure consistency in epidemiological studies.

Dysthymia, unlike recurrent depression, has maintained a consistent nosological status in all DSM and ICD versions since 1980. This likely explains the relatively greater number of epidemiological studies on dysthymia and persistent depression compared to recurrent depression [16-19]. However, even these studies are scarce compared to the extensive research on the nonspecific construct of major depressive disorder [16-19].

All the aforementioned characteristics of mood disorder taxonomy have directly influenced the disproportionately small number of basic and clinical studies on recurrent and persistent depression [47]. In this regard, when planning the design of future scientific studies, the following recommendations should be considered to improve the quality of the data obtained:

- **Separate verification of patients with single and recurrent depressive episodes:** Patients with a single depressive episode and those with recurrent depression should be verified separately. Given that definitions of recurrence may evolve, it is important to record the actual number of depressive episodes since the onset of the mood disorder, taking into account the duration of remission periods.
- **Recording duration of depressive episodes:** For patients in remission, the duration of the current depressive episode and/or the duration of the longest past depressive episode should be recorded to identify persistent depression, regardless of the polarity of the mood disorder.
- **Verification of dysthymia diagnosis:** The diagnosis of dysthymia should be verified in studies investigating any form of depression.
- **Sex-specific data on prevalence and risk factors:** Data on the prevalence and risk factors of dysthymia and all forms of depression should be provided separately for men and women, considering the sex disparities in the distribution of mood disorders.
- **Recording additional specifiers:** Additional specifiers for mood disorder episodes should be recorded, including age of onset, duration of the illness, subtype of depression according to DSM/ICD, and family history of mood disorders.

## 5 STUDY LIMITATION

This study has several limitations. First, despite searching well-known databases and existing meta-analyses, we may have missed some epidemiological studies on the lifetime prevalence of recurrent and persistent depression. Second, not all studies provided absolute numbers or percentages for prevalence, leading to discrepancies and potential inaccuracies when calculating missing data independently. Third, the evolving concept of bipolar disorders from DSM-III to DSM-5-TR may have influenced our findings. The inclusion of bipolar II disorder in DSM-IV (1994), changes in diagnostic criteria for mixed episodes, and the recognition of treatment-emergent hypomania/mania in DSM-5 (2013) have expanded the bipolar spectrum. As a result, some cases previously classified as unipolar depression may now be diagnosed as bipolar disorders. This shift could explain the observed decrease in the prevalence of unipolar depression and the increase in bipolar disorders. Fourth, the majority of epidemiological studies included in this review were conducted in North America and Western Europe, with limited representation from other regions. This geographical imbalance raises concerns about the generalizability of our findings to underrepresented populations. Regional differences in diagnostic practices, cultural perceptions of mental health, and accessibility to mental health care may significantly influence the reported prevalence rates of recurrent and persistent depression. For example, variations in the application of diagnostic criteria (e.g., DSM vs. ICD) and disparities in mental health infrastructure could lead to underdiagnosis or misclassification in certain regions. These factors highlight the need for more diverse and globally representative studies to ensure the validity and applicability of prevalence estimates across different populations.

## CONCLUSION

Despite the critical importance of recurrent depression, only three epidemiological studies from Switzerland, the USA, and Hungary exist, contrasting sharply with the greater number of studies on dysthymia and persistent depression. The underrepresentation of recurrent depression is partly attributed to the DSM's dominant role in psychiatric diagnostics. The broad diagnostic construct of major depressive disorder, criticized for its lack of proven validity, encompasses a wide spectrum of depressive conditions, diluting the focus on recurrent episodes. Moreover, persistent depression gained distinct status only in DSM-5, while recurrent depression still lacks diagnostic independence.

Our findings highlight the urgent need for refined diagnostic criteria and more comprehensive epidemiological studies. Future research should separately identify and verify recurrent and persistent depression, consider sex differences, and record detailed specifiers of mood disorder episodes. These improvements would enhance the accuracy and relevance of research, inform targeted mental health policies, and ensure appropriate resource allocation for individuals with recurrent depression. Clinically, refining diagnostic criteria and advancing epidemiological research could lead to more personalized treatment strategies, improving patient outcomes and reducing the long-term burden on healthcare systems.

## Figures and Tables

**Table 1:** Epidemiological studies of the lifetime prevalence of recurrent depression.

**Reference**	**Country**	**Diagnostic Methods**	**Sample Size**	**Prevalence**	**Sex Proportions Among the Cases**		**Note**
					**Female**	**Male**	
Vandeleur CL *et al.*, 2017 [21]	Switzerland	DSM-5	3720	10.5% (391)	69.1% (270)	30.9% (121)	Major depressive disorder total = 27.9%. Excluding the categories of persistent depressive disorder with persistent major depressive episode and persistent depressive disorder with intermittent major depressive episode.
Kessler RC, *et al.*, 2013 [22]	USA	DSM-IV‐TR	12175	10.3%* (~1254)	68.5% (~877)	31.5% (~404)	Major depressive disorder total = 14.4%, bipolar disorder total = 2.5%
Szádóczky E, *et al.*, 1998 [23]	Hungary	DSM-III-R	2953	10.3% (303)	75.9% (230)	24.1% (73)	Major depressive disorder total = 15.1%, bipolar disorder total = 5.1%

*The author’s absolute number of recurrent depression cases in the entire sample does not match the sum of the absolute number of recurrent depression cases in men and women (entire sample n=1254 *vs.* women + men n=1281). Therefore, the proportions of men and women with recurrent depression were calculated from the sum n=1281.

**Table 2:** Epidemiological studies of the lifetime prevalence of dysthymia, persistent and intermittent depressions.

**Reference**	**Country**	**Diagnostic Methods**	**Diagnosis**	**Sample Size**	**Prevalence**	**Sex Proportions Among the Cases**	
						**Female**	**Male**
Nübel J, *et al.*, 2020 [35]	Germany	DSM-IV-TR, CIDI	Persistent depression	4408	4.1% (179)	NA	NA
Khazaie H, *et al.*, 2019 [34]	Iran	DSM-IV-TR, CIDI	Dysthymia	2991	1.9% (57)	60.0% (34)	40.0% (23)
Vandeleur CL *et al.*, 2017 [21]	Switzerland	DSM-5	Dysthymia	3720	2.5% (92)	64.1% (59)	35.9% (33)
			Persistent depression		18.0% (670)	67.5% (452)	32.5% (218)
Murphy JA, Byrne GJ., 2012 [6]	Australia	DSM-5	Persistent depression and dysthymia	8841	4.5% (398)	65.8% (262)	34.2% (136)
			Persistent depression		~1.6% (144)	NA	NA
			Dysthymia		~2.9% (254)	NA	NA
Blanco C. *et al.*, 2010 [28]	USA	DSM-IV	Persistent depression	43093	3.2% (1377)	70.2% (~967)	29.8% (~410)
			Dysthymia		1.1% (456)	60.0% (~274)	40.0% (~182)
Baune BT, *et al.*, 2006 [33]	Germany	DSM-IV, CIDI	Dysthymia	4181	4.5% (~188)	65.0% (~122)	35.0% (~66)
Kessler RC, *et al.*, 2005 [27]	USA	DSM-IV(SCID)/WMH-CIDI	Dysthymia	9282	2.5% (~232)	NA	NA
Riolo SA, *et al.*, 2005 [29]	USA	DSM-III-R	Dysthymia	8446	6.1% (~515)	NA	NA
Jonas BS, *et al.*, 2003 [31]	USA	DSM-III-R	Dysthymia	7667	6.2% (475)	NA	NA
			Intermittent depression		3.4% (261)	NA	NA
Bijl RV, *et al.*, 1998 [26]	Netherlands	DSM-III-R, CIDI	Dysthymia	7076	6.3% (~446)	58.1% (~259)	41.9% (~187)
Takeuchi DT, *et al.*, 1998 [30]	USA	DSM-III-R	Dysthymia	1747	5.2% (91)	NA	NA
Carta MG, *et al.*, 1995 [32]	Italy, France	DSM-III-R	Dysthymia	480	4.1% (~20)	65% (~13)	35% (~7)
Kessler RC, *et al.*, 1994 [25]	USA	DSM-III-R, CIDI	Dysthymia	8098	6.4% (~518)	63.1% (~327)	36.9% (~191)
Wittchen HU, *et al.*, 1992 [37]	Germany	DSM-III	Dysthymia	483	~ 4.3% (21)	NA	NA
Wells JE, *et al.*, 1989 [38]	New Zealand	DSM-III	Dysthymia	l498	6.4% (96)	NA	NA
Weissman MM, *et al.*, 1988 [24]	USA	DSM III	Dysthymia	18248	3.1% (~566)	69.1% (~391)	30.9% (~175)
Szádóczky E,, *et al.*, 1998 [23]	Hungary	DSM-III-R	Dysthymia	2953	4.2% (124)	75.8% (~94)	29.0% (~36)
Bland RC, *et al.*, 1988 [36]	Canada	DSM-III	Dysthymia	3258	~4.4% (142)	NA	NA

## Data Availability

The data and supportive information are available within the article.

## LIST OF ABBREVIATIONS

ICD: = International Classification of Diseases
DSM: = Diagnostic and Statistical Manual of Mental Disorders

## STANDARDS OF REPORTING

PRISMA guidelines and methodology were followed.

## SUPPLEMENTARY MATERIAL

PRISMA checklist is available as supplementary material on the publisher’s website along with the published article.
